# Development of a deep learning algorithm for detecting significant coronary artery stenosis in whole-heart coronary magnetic resonance angiography

**DOI:** 10.1016/j.jocmr.2025.101932

**Published:** 2025-06-30

**Authors:** Masafumi Takafuji, Masaki Ishida, Takuma Shiomi, Ryohei Nakayama, Miyuko Fujita, Shintaro Yamaguchi, Yuzo Washiyama, Motonori Nagata, Yasutaka Ichikawa, Katsuhiro Inoue, Satoshi Nakamura, Hajime Sakuma

**Affiliations:** a Department of Radiology, Mie University Hospital, 2-174 Edobashi, Tsu, Mie 514-8507, Japan; b Department of Science and Engineering, Ritsumeikan University, 1-1-1 Noji-Higashi, Kusatsu, Shiga 525-8577, Japan

**Keywords:** Coronary artery disease, Coronary magnetic resonance angiography, Deep learning, Convolutional neural network, Invasive coronary angiography

## Abstract

**Background:**

Whole-heart coronary magnetic resonance angiography (CMRA) enables noninvasive and accurate detection of coronary artery stenosis. Nevertheless, the visual interpretation of CMRA is constrained by the observer's experience, necessitating substantial training. The purposes of this study were to develop a deep learning (DL) algorithm using a deep convolutional neural network to accurately detect significant coronary artery stenosis in CMRA and to investigate the effectiveness of this DL algorithm as a tool for assisting in accurate detection of coronary artery stenosis.

**Methods:**

Nine hundred and fifty-one coronary segments from 75 patients who underwent both CMRA and invasive coronary angiography (ICA) were studied. Significant stenosis was defined as a reduction in luminal diameter of >50% on quantitative ICA. A DL algorithm was proposed to classify CMRA segments into those with and without significant stenosis. A four-fold cross-validation method was used to train and test the DL algorithm. An observer study was then conducted using 40 segments with stenosis and 40 segments without stenosis. Three radiology experts and three radiology trainees independently rated the likelihood of the presence of stenosis in each coronary segment with a continuous scale from 0 to 1, first without the support of the DL algorithm, then using the DL algorithm.

**Results:**

Significant stenosis was observed in 84 (8.8%) of the 951 coronary segments. Using the DL algorithm trained by the four-fold cross-validation method, the area under the receiver operating characteristic curve (AUC) for the detection of segments with significant coronary artery stenosis was 0.890, with 83.3% sensitivity, 83.6% specificity, and 83.6% accuracy. In the observer study, the average AUC of trainees was significantly improved using the DL algorithm (0.898) compared to that without the algorithm (0.821, p < 0.001). The average AUC of experts tended to be higher with the DL algorithm (0.897), but not significantly different from that without the algorithm (0.879, p = 0.082).

**Conclusion:**

We developed a DL algorithm offering high diagnostic accuracy for detecting significant coronary artery stenosis on CMRA. Our proposed DL algorithm appears to be an effective tool for assisting inexperienced observers to accurately detect coronary artery stenosis in whole-heart CMRA.

## Graphical abstract

**Figure:**
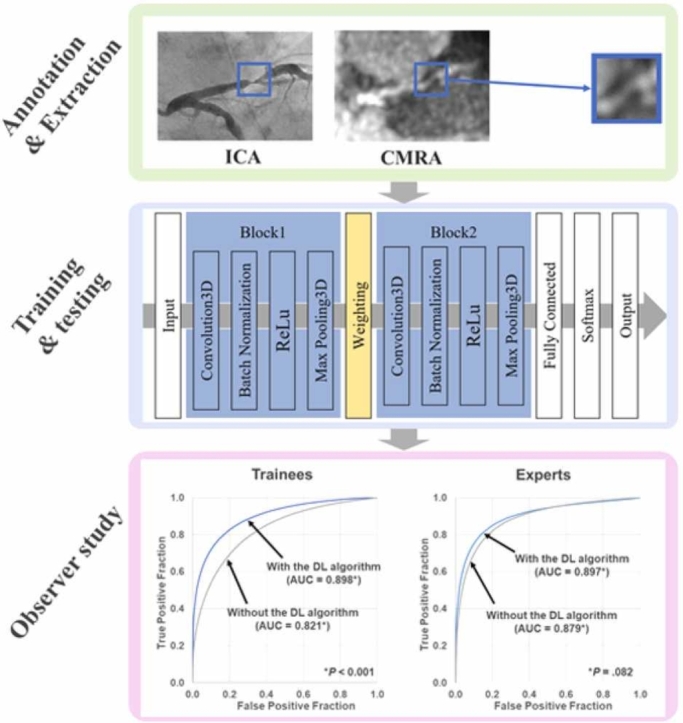

## 1 Introduction

Coronary artery disease (CAD) is a leading cause of morbidity and mortality worldwide [1]. Invasive coronary angiography (ICA) is currently regarded as the gold standard for detecting CAD, but is expensive, potentially invasive, and associated with a small risk of serious complications [2]. Coronary computed tomography angiography (CCTA) is a noninvasive alternative to ICA for ruling out CAD. However, CCTA shows several challenges in the assessment of vessels with heavily calcified plaque [3] and the need for radiation exposure [4], [5].

Whole-heart coronary magnetic resonance angiography (CMRA) enables accurate, noninvasive detection of coronary artery stenosis without the use of ionizing radiation. In addition, CMRA can visualize the lumen of the coronary artery in patients with severe coronary artery calcification [6]. Several single-center studies and a multicenter study have demonstrated good diagnostic performance from whole-heart CMRA for detecting significant coronary artery stenoses on ICA [7], [8], [9], [10], [11]. However, the visual interpretation of CMRA relies on the signal intensity profile along the coronary artery, requiring observer experience [12].

Recent advances in artificial intelligence technology, particularly deep learning (DL), have substantially improved medical image analysis [13], [14], [15], [16]. In recent years, DL algorithms using deep convolutional neural networks (DCNNs) have been employed in CCTA to identify coronary stenosis, which supports clinicians by enhancing the diagnostic performance for CAD [17], [18], [19], [20], [21], [22], [23], [24], [25]. However, no DL algorithm has yet been specifically designed for detecting coronary artery stenosis in whole-heart CMRA.

The purposes of this study were to develop a DL algorithm using a DCNN to accurately detect significant coronary artery stenosis in CMRA and to investigate the effectiveness of this DL algorithm as an assistance tool for accurately detecting coronary artery stenosis.

## 2 Materials and methods

### 2.1 Patient population

All study protocols were approved by the institutional review board (approval no. H2020-182). The need to obtain individual consent was waived based on the retrospective design.

Data were obtained from 75 patients (52 males; mean age, 65.7 ± 13.3 years) who underwent both CMRA using a 3.0T magnetic resonance (MR) scanner and ICA within 90 days without any intervention between examinations between May 2012 and January 2019. Median interval between ICA and CMRA was 41 days (interquartile range, 17–56 days). No patient had severe renal failure (glomerular filtration rate <30 mL/m^2^) or known allergies to contrast media. Exclusion criteria were arrhythmia at the time of cardiovascular magnetic resonance (CMR) or poor overall image quality due to severe artifacts. However, no patient met any exclusion criteria. The background characteristics of patients are shown in Table 1. To provide clinical context for the observed prevalence of coronary stenoses, we calculated the pre-test probability of obstructive CAD for all patients [26], [27]. Exactly 89% (67/75) of the patients in our cohort were classified as having an intermediate or high pre-test probability of obstructive CAD.

**Table 1:** Patient background.

	n = 75
Male, n (%)	52 (69)
Age (years), mean ±SD	65.7±13.3
Body mass index (kg/m^2^), mean±SD	24.4±3.8
Hypertension, n (%)	50 (67)
Dyslipidemia, n (%)	34 (45)
Diabetes mellitus, n (%)	21 (28)
Smoking, n (%)	40 (53)
Family history of CAD, n (%)	9 (12)
Post-PCI, n (%)	20 (27)

Data are numbers (%) of cases, means ± standard deviation*. SD* standard deviation, *CAD* coronary artery disease, *PCI* percutaneous coronary intervention

### 2.2 Cardiovascular magnetic resonance imaging

CMR studies were performed using a 3.0T MR scanner (Ingenia 3.0T; Philips Medical Systems, Best, the Netherlands) equipped with dS torso coils for signal reception. CMRA was performed as part of a comprehensive CMR protocol, including cine CMR, stress and rest perfusion CMR, and late gadolinium enhancement (LGE)-CMR. For both stress and rest perfusion CMR, gadoterate meglumine (Gd-DOTA; Guerbet Japan, Tokyo, Japan) was injected at a dose of 0.03 mmol/kg. LGE-MRI was performed 5–10 min after intravenous administration of Gd-DOTA, with a cumulative dose of 0.15 mmol/kg. Whole-heart CMRA images were acquired approximately 15 min after the completion of LGE-MRI, without any additional contrast injection. Five milligrams of isosorbide dinitrate were administered sublingually to all subjects before CMRA acquisition. Beta-blockers were not used. To monitor motions of the right coronary artery (RCA), trans-axial cine CMR was performed under free breathing for 50 cardiac phases. A patient-specific acquisition window in the cardiac cycle was set during either systole or diastole, depending on the phase of minimal motion of the RCA [11]. Free-breathing, navigator-gated three-dimensional whole-heart CMRA was obtained with a turbo field echo sequence using T2 preparation, fat saturation, and radial phase encoding [28] (repetition time, 3.7 ms; time to echo, 1.7 ms; flip angle, 15°; full Fourier encoding; excitations per cardiac cycle, 9–14; field of view, 330 × 280 mm; acquisition matrices, 256 × 193; reconstruction matrices, 512 × 512; acquisition slice thickness, 1.6 mm; reconstruction slice thickness, 0.8 mm; sensitivity encoding factor, 3.3) [29], [30]. Slab thickness was adapted for each patient to cover the entire heart. Additional motion tracking was not employed. The navigator gating window was ± 2.5 mm. Heart rate was measured using the electrocardiogram employed for gating during CMRA imaging.

### 2.3 Invasive coronary angiography

ICA was performed using the standard Judkins technique [31]. ICA was interpreted by one experienced observer (K.I.) who was blinded to all other studies. Significant CAD was defined as a ≥50% reduction in luminal diameter on quantitative ICA (Kada-View; Photron Medical Imaging, Tokyo, Japan).

### 2.4 Datasets

**Fig. 1:**
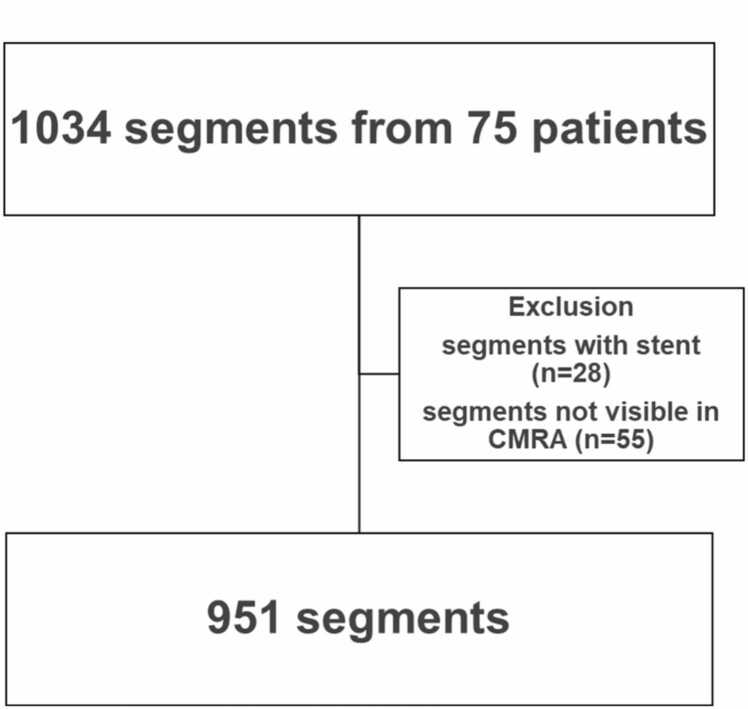
Overview of the study design and patient selection protocol. *CMRA* coronary magnetic resonance angiography

**Fig. 2:**
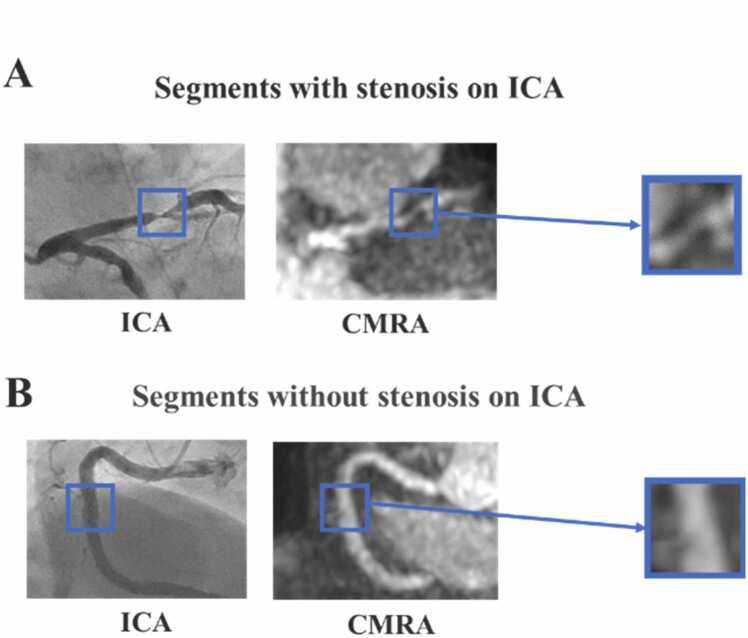
Segment annotation. Segments with stenosis on ICA were identified on CMRA and annotated at the stenotic lesion (A). Segments without stenosis on ICA were annotated at a representative site on CMRA images (B). VOIs of 21 × 21 × 21 pixels centered on the annotated points were extracted from the CMRA image. *ICA* invasive coronary angiography, *CMRA* coronary magnetic resonance angiography, *VOI* volume of interest

Coronary arteries were divided into 18 segments according to Society of Cardiovascular Computed Tomography (SCCT) guidelines [32]. Among 1034 segments from 75 patients who were visible on ICA, 28 segments with stents and 55 segments not visible on CMRA were excluded. The resulting dataset comprised 951 segments (Fig. 1). Segments with stents were excluded because substantial imaging artifacts associated with stented segments in CMRA inherently limit the accurate evaluation of stenosis in these regions. The number and proportion of segments with each level of stenosis are shown in Table 2. Segments with significant stenosis on ICA were identified on CMRA images and annotated as stenotic lesions by an experienced radiologist (M.T., 8 years of experience with CMR). Segments without significant stenosis on ICA were annotated at a representative point on each SCCT segment (Fig. 2) [32]. Volume of interests (VOIs) of 21 × 21 × 21 pixels centered on the annotated points were extracted from the CMRA image. Signal values in VOIs were normalized by dividing them by the average signal value of the CMRA images for the corresponding patient. The proposed method was developed and evaluated using MATLAB (MathWorks, Inc., Natick, Massachusetts) on a workstation (central processing unit: Intel Core i7-17900F processor; random-access memory: 32 GB; graphics processing unit: NVIDIA RTX 3060, NVIDIA, Santa Clara, Californa).

**Table 2:** The number and proportion of segments with each level of stenosis.

	All datasets (n = 951)	Observer study (n = 80)
0–24%, n (%)	852 (89.6)	40 (50.0)
25–49%, n (%)	15 (1.6)	0 (0.0)
50–69%, n (%)	37(3.9)	17 (21.3)
70–99%, n (%)	40(4.2)	20 (25)
100%, n (%)	7 (0.7)	3 (3.8)
Stent, n	28	

Data are numbers (%) of cases, means ± standard deviation.

### 2.5 Structure of the DL algorithm

**Fig. 3:**
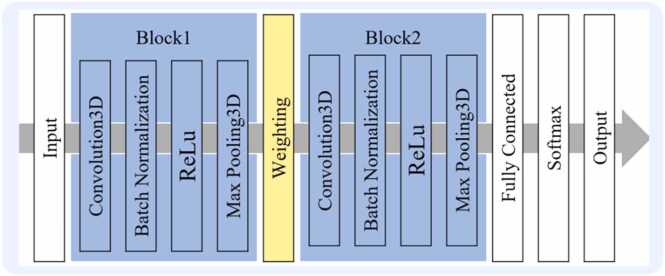
The DL algorithm comprised two feature-extracting blocks with a weighting layer, as well as a separate fully connected layer. Each feature-extracting block consisted of a 3D convolution layer, a batch normalization layer, a rectified linear unit (ReLU) activation function, and a max pooling layer. *3D* three-dimensional, *DL* deep learning

The DL algorithm comprised two feature-extracting blocks, a weighting layer, and a classifier. Fig. 3 illustrates the overall schema. Each feature-extracting block included a convolution layer, a batch normalization layer, a rectified linear unit (ReLU) function, and a max pooling layer. A VOI with 21 × 21 × 21 voxels from the CMRA image was input into the network. Feature-extracting block 1 generated feature maps of 11 × 11 × 11 voxels. In the weighting layer, feature maps at the center of the VOI were weighted by the Hadamard product with a Gaussian-distributed weight matrix that had an empirically determined standard deviation (SD) of 0.75. These weighted maps were then fed into the feature-extracting block 2. The 6 × 6 × 6 feature maps obtained from feature-extracting block 2 were processed through a fully connected layer, a softmax function, and a classification layer, resulting in an output for the probability of the input VOI having significant stenosis.

### 2.6 Training and testing of the DL algorithm

The DL algorithm was trained and tested using a four-fold cross-validation method based on patient data. To ensure that the number of segments with significant stenosis was approximately equal, patient data were divided into four subsets. One subset was used as test data, two subsets as training data, and the remaining one as validation data. The DL algorithm was trained and evaluated four times until each of the four subsets had been used as the test dataset once. VOIs with significant stenosis were rotated 90° along each axis, augmenting the number of data four-fold. The loss function for training was the weighted cross-entropy error (WCEE):

(1) $$\mathrm{WCEE}=\;-\frac{1}{n}\sum_{i\;=\;1}^{n}w_{i}t_{i}\log\left(y_{i}\right)$$

where n is the number of training data, $w_{i}$ is the class weight, $t_{i}$ is the true class vector, and $y_{i}$ is the predicted class vector. Hyperparameters included a batch size of 4, a maximum of 50 epochs, and a learning rate of 1e−4. Stochastic gradient descent with a momentum of 0.9 was used to optimize network weights [33]. Here, the early stopping was applied with the validation data.

### 2.7 Observer study

**Fig. 4:**
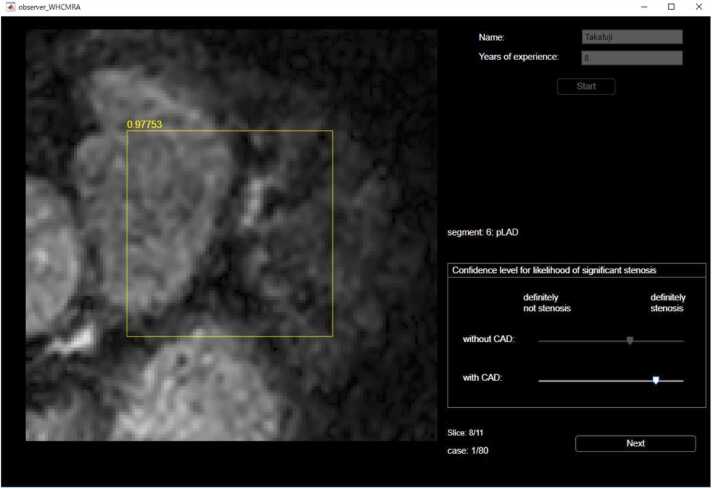
Dedicated user interface used for image evaluation. A 128 × 128 trans-axial region of interest centered on the target coronary segment is displayed, with 11 contiguous image slices (1 mm thickness) available for scrolling. The interface supports adjustment of window width and level and displays the target segment number to guide consistent evaluation. *CAD* coronary artery disease

An observer study was conducted to investigate the efficacy of the DL algorithm as a supportive tool for two groups of observers based on levels of experience in the diagnosis of CAD by CMRA, i.e., three trainees with less than 2 years of CMR experience (Y.W., S.Y., and M.F.) and three experts with more than 15 years of CMR experience (M.I., M.N., and Y.I.) participated in this observer study. The observers independently rated the likelihood of stenosis presence in each coronary segment using a continuous scale from 0 to 1, where 0 indicates a 0% likelihood of stenosis and 1 indicates a 100% likelihood of stenosis. The observers initially made assessments without the support of the DL algorithm. Then, after obtaining probability scores from the DL algorithm, the likelihood of the presence of stenosis was reassessed. During the evaluation, a dedicated user interface was used to present the image data. For each case, a trans-axial 128 × 128 region of interest centered on the target coronary segment was displayed. Observers were able to scroll through 11 contiguous image slices with 1 mm thickness to examine the segment in detail. The interface allowed adjustment of the window width and level to optimize image contrast. Additionally, the target segment number was displayed to ensure clarity and consistency in the evaluation (Fig. 4). A total of 80 segments were selected from the full dataset of 951 segments using a stratified random sampling method based on the probability scores generated by the DL algorithm. These scores were generated from a model that did not include those segments in its training. These included 40 segments with significant stenosis and 40 segments without significant stenosis. These segments were selected to ensure the area under the receiver operating characteristic curve (AUC) for these segments was approximately equal to that for all 951 segments. The number and proportion of segments with each level of stenosis are shown in Table 2.

### 2.8 Statistical analysis

Normality of continuous variables was assessed using the Shapiro-Wilk test. As all continuous variables were normally distributed, data for continuous variables are presented as the mean ± SD. Categorical variables are presented as frequencies and percentages.

If the probability output by the DL algorithm exceeded a threshold, the VOI was classified as having significant stenosis. The threshold value was set to maximize the Youden index based on receiver operating characteristic (ROC) analysis of test data. Classification accuracy, sensitivity, and specificity were evaluated based on the probability of significant stenosis. AUCs were calculated to evaluate the classification accuracy of the proposed DL algorithm and the diagnostic performance of the observer with and without the DL algorithm. The Delong test was used to compare AUCs. All statistical analyses were performed using R version 4.3.2 (R Foundation for Statistical Computing, Vienna, Austria). Values of p < 0.05 were considered statistically significant.

## 3 Results

Mean heart rate during CMRA scanning was 76 ± 12 beats/min. The mean data acquisition window for CMRA was 50 ± 23 ms (acquisition in systole 61%, diastole 39%). Among the 951 segments, 84 segments (8.8%) exhibited significant stenosis as defined by ICA, while 867 segments did not.

### 3.1 Classification accuracy of the DL algorithm for significant coronary artery stenosis

**Fig. 5:**
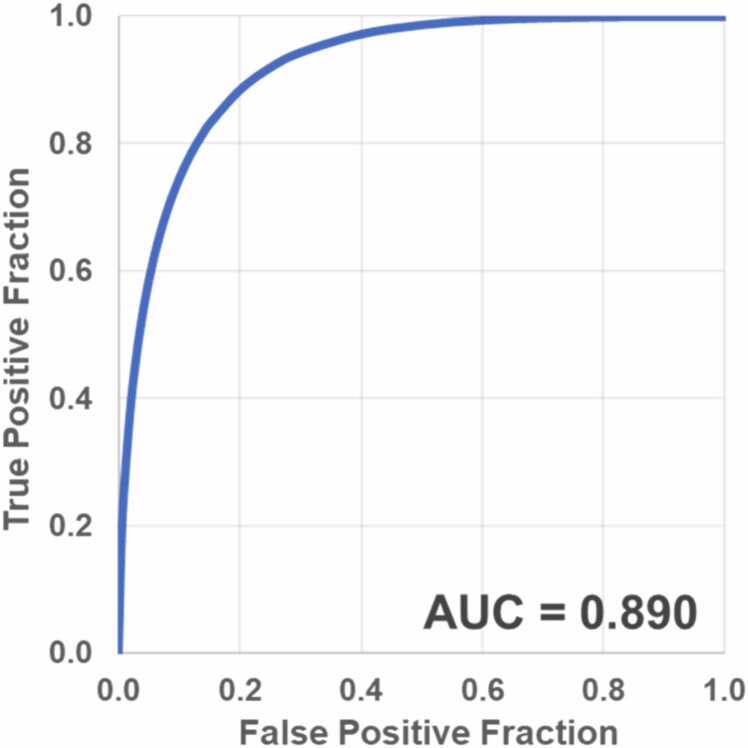
Receiver operating characteristic curves in detecting significant coronary artery stenosis for the DL algorithm. *AUC* area under the curve, *DL* deep learning

Using the DL algorithm, the AUC for detecting segments with significant coronary artery stenosis was 0.890 (Fig. 5). The sensitivity, specificity, and accuracy were 83.3%, 83.6%, and 83.6%, respectively.

### 3.2 Observer study

**Fig. 6:**
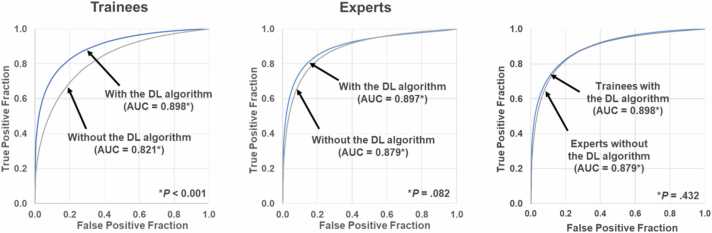
Averaged receiver operating characteristic curves in detecting significant coronary artery stenosis with and without the support of the DL algorithm for the three trainee observers and three expert observers. *AUC* area under the receiver operating characteristic curve, *DL* deep learning

In the observer study, the average AUC for trainees without DL algorithm support (0.821) was significantly lower than that of experts (0.879, p = 0.029). AUCs improved for five of the six observers when using our DL algorithm. The average AUC of trainees with the support of the DL algorithm (0.898) was significantly higher than that without the support of the DL algorithm (0.821, p < 0.001) (Fig. 6). The average AUC of experts with the support of the DL algorithm (0.897) tended to be higher than that without support (0.879, p = 0.082), but this difference was not significant. No significant differences were identified between the average AUC of trainees with DL algorithm support and that of experts without DL algorithm support (p = 0.432). Similarly, no significant difference was observed between the average AUC of trainees with DL algorithm support and that of experts with DL algorithm support (p = 0.971).

## 4 Discussion

The main findings of this study were as follows: 1) the DL algorithm, which was specifically developed for detecting coronary artery stenosis on whole-heart CMRA images, showed good classification accuracy when used to identify significant coronary artery stenosis confirmed by ICA; and 2) the diagnostic performance of trainees improved significantly with the support of the DL algorithm. The performance of experts also tended to improve with DL algorithm support, although this improvement was not significant. These findings highlight the potential for routine clinical use of DL-supported CMRA tools for detecting obstructive CAD in a real-world setting.

To the best of our knowledge, this represents the first study to develop a DL algorithm specifically designed to detect coronary artery stenosis in whole-heart CMRA. Whole-heart CMRA provides distinct advantages over CCTA, including robustness to heavy coronary calcification and the absence of radiation exposure [6]. However, CMRA has lower spatial resolution than CCTA, necessitating visual assessment of the signal intensity profile along the coronary artery to diagnose coronary artery stenosis, rather than simply relying on the reduction in lumen diameter for the coronary artery [12]. This reliance on visual interpretation makes CMRA diagnosis heavily reliant on observer experience and necessitates extensive training. Diagnostic support tools to aid in the interpretation of CMRA are thus clearly needed. DL algorithms have shown marked success in detecting objects and regions within images across various fields [13], [14], [15], [16]. In medical imaging, DL techniques have demonstrated diagnostic accuracy comparable to that of human experts [34]. In addition, some studies have validated the utility of DL in the detection of CAD using CCTA and ICA [17], [18], [19], [20], [21], [22], [23], [24], [25]. The achievements in the current study highlight the potential for DL to significantly enhance the diagnostic process for CMRA.

In our study, the proposed deep DL algorithm for CMRA demonstrated comparable diagnostic performance to expert visual assessment for detecting CAD, achieving 83.7% sensitivity, 83.3% specificity, and 83.7% accuracy among 951 coronary segments, with an 8.8% prevalence of stenosis. The diagnostic performance of the model may vary depending on the stenosis threshold used. In this study, a 50% cut-off was applied, consistent with widely accepted clinical and research standards. For comparison, a multicenter study by Kato et al. [10] using whole-heart CMRA at 1.5T visually evaluated 1461 coronary segments from 127 patients with suspected CAD. That study reported a 6.2% prevalence of stenosis and achieved 81% sensitivity, 98% specificity, and 97% accuracy for detecting ≥50% stenosis on ICA. Similarly, a single-center study using a 3T MR system visually examined 781 segments from 62 patients with a 12.2% prevalence of stenosis, and reported 91.6% sensitivity, 83.1% specificity, and 84.1% accuracy for whole-heart CMRA in identifying significant stenosis [9]. The findings of our study underscore the potential of DL algorithms as reliable diagnostic tools for the noninvasive assessment of CAD using CMRA.

The diagnostic accuracy of our proposed DL algorithm for CMRA was also comparable to that reported in studies investigating DL-based approaches for CCTA for diagnosing CAD as defined by ICA [23], [24], [25]. Han et al. demonstrated that the DL algorithm for CCTA exhibited 88% sensitivity, 85% specificity, and 86% accuracy, with an AUC of 0.870 in detecting significant CAD [24]. The same research group also conducted a multicenter study to externally validate the DL algorithm across subgroups stratified by sex, age, geographic area, and computed tomography (CT) scanner type [25]. This study reported 65.7% sensitivity and 85.6% specificity, with AUC values ranging from 0.79–0.88 across various patient subgroups and CT systems. The diagnostic performance of DL algorithms for CCTA can be compromised by beam-hardening artifacts caused by high-density calcium in the original CT images. A previous CCTA study [35] showed that the accuracy of a DL algorithm was lower (85.7%) in patients with severe calcification compared to those with mild calcification at the segment level (92.8%). Considering the overall comparable diagnostic performance of DL algorithms for CMRA and CCTA, the DL approach for CMRA may offer advantages in cases of heavily calcified lesions.

The DL algorithm should not only offer sufficient classification accuracy but also demonstrate effectiveness as a supportive tool for inexperienced and experienced observers in clinical diagnosis. In the current study, the diagnostic performance for detecting significant coronary stenosis improved with the support of our proposed algorithm. Notably, with the support of the DL algorithm, the diagnostic performance of trainees with less than 2 years of experience in CMR significantly improved the diagnostic accuracy to a level comparable to that of experts. These findings align with the results of a previous study using CCTA, which investigated the impact of DL algorithm support on the diagnostic performance of observers with varying levels of experience [35]. This CCTA study [35] demonstrated that the support of DL algorithms enhanced the diagnostic performance of readers across all experience levels, with the most notable benefits observed among observers with less experience. The integration of these DL algorithms into clinical settings has the potential to standardize and elevate diagnostic accuracy by bridging the skill gap for less experienced readers.

## 5 Limitations

This study has several limitations. First, this was a retrospective study conducted using data from a small cohort at a single institute. The limited dataset size raises concerns about potential overfitting during the training of the DL algorithm, which may affect the generalizability of the findings. To mitigate this risk, we employed early stopping based on validation data to enhance model generalizability. Second, while the current model classifies stenosis using a binary threshold of 50%, clinical decision-making often requires more granular stratification (e.g., 50%, 75%, and 100% stenosis). Technically, our framework could be extended to support multi-class classification. However, due to the limited number of cases, it was not feasible to develop or validate a reliable multi-class algorithm in this study. Larger multicenter studies are warranted to support the development of a clinically meaningful multi-class stenosis classification model. Third, the observer study included a relatively small number of segments (n = 80), which may limit the statistical power of the ROC analysis. This sample size was selected to maintain a reasonable reading time (approximately 40 min) for each observer, in line with established recommendations to minimize fatigue during image interpretation [36]. Additionally, the segments used in the observer study were selected from the same dataset of 951 coronary segments that had been used for training and testing the DL algorithm. This overlap may compromise the validity of the performance assessment due to potential bias. Validation using an entirely independent external dataset is necessary to provide evidence of generalizability. Fourth, the CMR images were collected between 2012 and 2019. Advances in scanner hardware and imaging protocols since then may influence both the performance and clinical applicability of the proposed method. Future validation studies using more contemporary external datasets are necessary to confirm the generalizability of our results. Fifth, although previous reports have described gadolinium leakage into atherosclerotic plaques and native T1 enhancement [37], [38], which may potentially affect the diagnostic performance of CMRA, we did not observe such findings in our dataset. In our previous CMRA studies, no cases of plaque enhancement were identified. Moreover, to our knowledge, no systematic investigations have assessed the impact of this phenomenon on CMRA interpretation. While we cannot completely rule out its influence, we believe its effect on the diagnostic assessment of coronary artery stenosis is likely minimal. Sixth, segments that were completely non-visible on CMRA, primarily corresponding to small distal vessels, were excluded from analysis due to the spatial resolution limitations of the modality. As such, the presence of stenosis in these non-visualized segments cannot be definitively excluded. While none of these segments exhibited significant stenosis on ICA, we acknowledge that the inability to visualize them on CMRA introduces a potential limitation. Seventh, a direct comparison between CMRA and CCTA was not performed in the present study. While CCTA remains a widely accepted clinical reference standard, especially for noninvasive coronary imaging, our discussion of the advantages of CMRA, such as reduced susceptibility to calcification-related artifacts, is based on theoretical assumptions and limited empirical evidence [39]. Direct comparative studies between MRA and CCTA in the same patient population are warranted to validate these theoretical advantages and clarify the clinical utility of CMRA in this context. Finally, segments with and without significant stenosis were manually annotated by an experienced radiologist, introducing variability that could have affected classification performance. However, we consider that the overall usefulness of the proposed method with attention mechanisms remains intact.

## 6 Conclusions

We developed a DL algorithm offering high diagnostic accuracy for detecting significant coronary artery stenosis on CMRA. Our proposed DL algorithm appears to provide an effective tool for assisting inexperienced observers to accurately detect coronary artery stenosis in whole-heart CMRA.

## Author contributions

**Masafumi Takafuji:** Writing – original draft, Visualization, Investigation, Formal analysis, Data curation. **Masaki Ishida:** Writing – review & editing, Methodology, Investigation, Formal analysis, Data curation, Conceptualization. **Takuma Shiomi:** Investigation, Formal analysis. **Ryohei Nakayama:** Writing – review & editing, Methodology, Investigation, Formal analysis. **Miyuko Fujita:** Investigation. **Shintaro Yamaguchi:** Investigation. **Yuzo Washiyama:** Investigation. **Motonori Nagata:** Investigation. **Yasutaka Ichikawa:** Investigation. **Katsuhiro Inoue:** Investigation. **Satoshi Nakamura:** Data curation. **Hajime Sakuma:** Writing – review & editing, Supervision, Project administration.

## Ethics approval and consent

This study was conducted in accordance with the principles of the Declaration of Helsinki and with the approval of the Mie University Institutional Review Board at Mie University Hospital (approval no. H2020-182). The need for written informed consent was waived because of the retrospective design.

## Consent for publication

Not applicable.

## Declaration of competing interests

The authors declare the following financial interests/personal relationships which may be considered as potential competing interests: Hajime Sakuma reports financial support was provided by JSPS KAKENHI (18K07749). Satoshi Nakamura reports a relationship with Siemens Healthcare K.K. that includes funding grants. The other authors declare that they have no known competing financial interests or personal relationships that could have appeared to influence the work reported in this paper.
